# Current Status and Future Directions of Proton Therapy for Head and Neck Carcinoma

**DOI:** 10.3390/cancers16112085

**Published:** 2024-05-30

**Authors:** Sara Lillo, Alfredo Mirandola, Alessandro Vai, Anna Maria Camarda, Sara Ronchi, Maria Bonora, Rossana Ingargiola, Barbara Vischioni, Ester Orlandi

**Affiliations:** 1 Radiation Oncology Unit, Clinical Department, National Center for Oncological Hadrontherapy (CNAO), 27100 Pavia, Italy; annamaria.camarda@cnao.it (A.M.C.); sara.ronchi@cnao.it (S.R.); maria.bonora@cnao.it (M.B.); rossana.ingargiola@cnao.it (R.I.); barbara.vischioni@cnao.it (B.V.); ester.orlandi@cnao.it (E.O.); 2 Medical Physics Unit, Clinical Department, National Center for Oncological Hadrontherapy (CNAO), 27100 Pavia, Italy; alfredo.mirandola@cnao.it (A.M.); alessandro.vai@cnao.it (A.V.); 3 Department of Clinical, Surgical, Diagnostic, and Pediatric Sciences, University of Pavia, 27100 Pavia, Italy

**Keywords:** head and neck cancer, oropharyngeal cancer, nasopharyngeal cancer, proton therapy, future directions

## Abstract

**Simple Summary:**

Proton therapy allows for more effective organs at risk avoidance than photon radiotherapy, thus reducing both radiation-induced toxicity and second cancer risk. The aim of the present paper is to compare protons and photons outcomes in the context of oropharyngeal and nasopharyngeal cancer and provide an updated comprehensive overview of the most promising new approaches and methodologies for treating head and neck cancer with protons.

**Abstract:**

The growing interest in proton therapy (PT) in recent decades is justified by the evidence that protons dose distribution allows maximal dose release at the tumor depth followed by sharp distal dose fall-off. But, in the holistic management of head and neck cancer (HNC), limiting the potential of PT to a mere dosimetric advantage appears reductive. Indeed, the precise targeting of PT may help evaluate the effectiveness of de-escalation strategies, especially for patients with human papillomavirus associated-oropharyngeal cancer (OPC) and nasopharyngeal cancer (NPC). Furthermore, PT could have potentially greater immunogenic effects than conventional photon therapy, possibly enhancing both the radiotherapy (RT) capability to activate anti-tumor immune response and the effectiveness of immunotherapy drugs. Based on these premises, the aim of the present paper is to conduct a narrative review reporting the safety and efficacy of PT compared to photon RT focusing on NPC and OPC. We also provide a snapshot of ongoing clinical trials comparing PT with photon RT for these two clinical scenarios. Finally, we discuss new insights that may further develop clinical research on PT for HNC.

## 1. Introduction

Radiation therapy (RT) plays a crucial role in the holistic management of head and neck cancer (HNC) both in the definitive and postoperative setting with or without systemic therapy. In particular, in patients with locally advanced nasopharyngeal cancer (NPC) and oropharyngeal cancer (OPC), the current standard approach involves a combination of RT and chemotherapy [1,2].

Over the past decades, several significant improvements have been made in the RT field enhancing precision and accuracy, essentially leading to an improvement in the profiles of both acute and late toxicity, with a beneficial effect on patients’ quality of life. So far, intensity modulated radiation therapy (IMRT) represents the state-of-the-art RT for HNC [3,4]. However, despite the dosimetric enhancements provided by IMRT, normal tissues inevitably receive exit doses from photons, potentially increasing the rate of side effects and the risk of secondary tumors. The issue of reducing toxicity burden is particularly emphasized when considering that approximately 59% of men and 62% of women with squamous cell carcinoma of head and neck (HNSCC) are alive 5 years after diagnosis [5] and the progressively increasing incidence of human papillomavirus (HPV)-associated OPC. Indeed, patients with HPV-associated OPC are relatively young and have a good prognosis; therefore, they are expected to live long enough to experience late toxicities that may have an impact on their quality of life (QoL) [6,7,8]. Moreover, most patients diagnosed with NPC are typically middle-aged adults in their prime, and dealing with late side effects is a challenge for those who survive [9].

In this scenario, the growing interest in proton therapy (PT) appears justified by the evidence that the dose distribution of protons allows maximal dose release at the tumor depth, followed by sharp distal dose fall-off, which is responsible for a more effective organs at risk (OARs) avoidance [10].

Data from the most updated survey on the current practice among European PT centers have shown that adult patients are subjected to PT if the main objective is toxicity risk decrease or, more rarely, dose escalation for increasing tumor control. In addition, younger and geriatric patients can benefit from PT as well, the former due to longer life expectancy, and the latter due to increased vulnerability [11]. However, beyond the toxicity reduction and QoL improvement primarily based on the ballistic properties of protons, other potential clinical implementations could arise from these properties, from radiobiological aspects or from the combination of the two.

In particular, the precise targeting of PT can help evaluate the effectiveness of volume reduction strategies while avoiding dose bias, especially for patients with HPV-OPC and NPC. Furthermore, PT could have potentially greater immunogenic effects than conventional photon therapy, possibly enhancing both the RT capability to activate anti-tumor immune response and the effectiveness of immunotherapy drugs [12]. These and other aspects are still relatively unexplored.

Based on these premises, the aim of the present paper is to conduct a narrative review reporting the safety and efficacy of PT compared to IMRT focusing on OPC and NPC. We also provide a snapshot of ongoing clinical trials testing PT for these two clinical scenarios. Finally, we discuss new insights that may further develop clinical research on PT for HNC.

## 2. Materials and Methods

To provide a comprehensive and up-to-date overview of the PT outcomes in terms of safety and efficacy compared to photon RT in patients with OPC and NPC, taking into account the heterogeneity of the literature and the specificity of the topic, a narrative review approach was chosen. Papers were retrieved from PubMed using the following search syntax: (“proton therapy” OR “proton radiotherapy” OR “proton radiation therapy” OR “intensity modulated proton therapy”) AND (“photon therapy” OR “photon radiotherapy” OR “photon radiation therapy” OR “intensity modulated radiotherapy” OR “volumetric modulated arc therapy”) AND (“nasopharyngeal cancer” OR “nasopharyngeal carcinoma” OR “oropharyngeal cancer” OR “oropharyngeal carcinoma”). No specific constraints on the type of study, year of publication, and number of subjects included were applied. Only articles in English were included in the study.

## 3. Results

### 3.1. Toxicity Reduction: Nasopharyngeal Cancer

Nasopharynx is undoubtedly the most challenging anatomical site to treat with RT due to the close proximity of important radiosensitive neurological structures. In this scenario, despite no randomized data being available to date, PT allows sparing of nearby OARs and therefore a better toxicity profile without affecting oncological outcomes [13]. According to the largest comparative analysis of curative-intent intensity-modulated proton therapy (IMPT) vs. IMRT for patients with nonmetastatic NPC, grade 3 mucositis (3.6% vs. 10.2%, *p* = 0.03), weight loss (0% vs. 10.2%, *p* < 0.001), and dysphagia (3.6% vs. 4.1%, *p* = 0.05) were significantly reduced in the IMPT group [14]; although there were no significant differences in terms of overall survival and progression-free survival, IMPT was found to be associated with decreased locoregional failure (*p* < 0.001).

Wu et al. have recently retrospectively compared the incidence and severity of chronic rhinosinusitis in 53 patients with NPC who underwent IMPT with 54 who underwent volumetric modulated arc therapy (VMAT) [15]; a modified Lund–Kennedy endoscopic scoring system and a Lund–Mackay staging score determined from MRI scans were used to evaluate the inflammation of the mucosa in the maxillary, anterior, and posterior ethmoid sinuses, observing significantly higher rates in the VMAT group and also persisting in the second post-RT year.

With regard to percutaneous endoscopic gastrostomy (PEG) tube placement, 2/10 IMPT-treated patients (20%) and 13/20 IMRT-treated patients (65%) required PEG after exclusive chemoradiation in a prospective observational study by Holliday et al. [16]. This result is probably consequent to the statistically significant lower mean doses to the oral cavity, as confirmed in a larger cohort by McDonald et al. in which PT was also associated with a reduced rate of opioid pain medication requirement [17] and in a more recent retrospective propensity score-matched analysis of 160 patients by Chou et al. [18]. The insertion of a PEG was necessary in only four patients (9.3%) in the retrospective analysis by Jiří et al. including 40 patients with NPC treated with IMPT, but another interesting finding of this study was that the most frequent grade 3 acute toxicity was radiation-induced dermatitis (RID) that occurred in six patients (14%). Indeed, according to Chou et al., PT seems to cause a significantly increased rate of grade 3 RID compared to VMAT (35% vs. 7.5%, *p* < 0.00) [18], as a consequence of the slightly higher entrance dose at the skin typical of protons; also, Williams et al. reported 11/26 (42%) cases of RID after IMPT, a price worth paying if we consider the 2-year locoregional control, freedom from distant metastasis, and overall survival rates of 92%, 87%, and 85%, respectively [19].

On the other hand, the advantage of PT in terms of central nervous system toxicities such as temporal lobe necrosis (TLN) is still a matter of debate. Liu et al. retrospectively reviewed 483 patients with HNC categorized into nasopharyngeal (198) and non-nasopharyngeal (285) groups [20]. As expected, the patients in the nasopharyngeal group experienced higher rates of TLN (5.6% vs. 0.4%, *p* < 0.01) especially in the early period after RT, but no significant differences were highlighted when comparing PT with VMAT (4.7% vs. 6.3%, *p* = 0.76).

### 3.2. Toxicity Reduction: Oropharyngeal Cancer

A significant dose reduction in the parotid was observed for IMPT compared with IMRT and 3D-CRT while keeping similar target coverage results [21,22,23]. Moreover, Bagley et al. reported the xerostomia-related quality of life (XeQoL) of 69 patients receiving a median IMPT dose of 69.3 Gy relative biological effectiveness (RBE) [24]; the highest scores reflecting a worse XeQoL were registered at 6 weeks during treatment, with a significant improvement starting 10 weeks after treatment and for the whole year afterwards. Even though some clinicodemographic risk factors such as continued smoking, female sex, and lower education are additional contributors for moderate-to-severe xerostomia regardless of the RT technique used [25], IMPT seems to be associated with reduced rates of xerostomia at 18–36 months after treatment [26].

Moreover, Blanchard et al. showed that IMPT is associated with a significant reduction in PEG tube placement with respect to IMRT [27]. Indeed, 1 year after the end of RT, PEG was present in 2% and 7.8% of patients subjected to IMPT and IMRT, respectively, with a weight loss that was three times lower in the IMPT cohort. These findings are further corroborated by the retrospective analysis by Manzar et al. in which PEG tube placement, hospitalization, and narcotic use rates are in favor of IMPT [28].

On the other hand, mandibular osteoradionecrosis (ORN) following PT is still a controversial issue. The incidence was estimated for the first time in a retrospective cohort of patients with OPC treated with definitive RT with or without concurrent chemotherapy at The University of Texas M.D. Anderson Cancer Center [29]. In detail, 534 patients received IMRT with a mean dose to the mandible of 41.2 Gy, and 50 patients received IMPT with a mean dose to the mandible of 25.6 Gy (RBE). ORN occurred in 41 patients (7.7%) of the IMRT group; in contrast, only one patient in the IMPT group (2%) developed ORN, showing that it was significantly associated with higher total dose and mandibular volume exposed to RT. Despite these promising results, a more recent and larger case series proved that ORN of the jaw remains a clinical challenge even in the era of highly conformal PT; of 122 patients treated at the Memorial Sloan Kettering Cancer Center, 13 (10.6%) developed ORN at a median follow-up time of 40.6 months, and the 3-year and 5-year rates of ORN were 5.2% and 11.5%, respectively [30].

In the last few years, an increasing number of studies focused on the possible translation of the dosimetric advantages of PT into measurable improvements in the QoL of patients with OPC, suggesting that it is responsible for less QoL deterioration than photon-based RT [31]. Sio et al. collected patient-reported outcomes data at baseline and in the acute, subacute, and chronic phases after treatment and found out that symptom burden was significantly lower among the patients that underwent IMPT, in particular dysgeusia and decreased appetite during the subacute and chronic phases [32]. Interestingly, QoL benefits were maintained even if PT was delivered in the postoperative setting [33].

Finally, in a recent study by Smith et al., work productivity and activity impairment questionnaires were administered to 147 patients with OPC at different time slots with respect to RT [34]. In general, 40% of patients receiving curative chemoradiation failed to return to work after treatment, but patients randomized to IMPT were more able to keep on working and demonstrated a favorable trend toward higher productivity recovery levels.

The ongoing clinical trials comparing PT to photon RT in patients with HNC that may provide clinicians with valuable evidence are summarized in Table 1.

### 3.3. Reduced Second Primary Malignancies

Among the potential sequelae of RT, the development of a second primary malignancy (SPM) is an event of particular relevance especially for patients with HNSCC who due to the advent of increasingly advanced therapies have a relatively high life expectancy. In their retrospective analysis, Ng et al. reported a prevalence of SPM of 9% in a cohort of 1512 patients with HNSCC treated with curative-intent RT, with a median time of development of 72 months and no significant difference between 3D-CRT and IMRT [35].

The recent study by Jain et al. focused on the predicted risk of SPM in a cohort of patients with HPV-positive OPC subjected to transoral robotic surgery (TORS) and selective neck dissection followed by adjuvant IMPT [36]. Both IMPT and IMRT plans were generated for each patient, and previously reported models of organ-specific radiation-induced cancer incidence were used to address the aim of the study. Although both RT techniques provided good target coverage, a lower mean organ equivalent dose was recorded with protons, resulting in a significant decrease in the relative risk of SPM of 4 patients out of 100 per year.

Consistent with these results, PT was associated with a significantly lower risk of SPM compared with IMRT among 450,373 patients divided into nine tumor categories and followed up for a median time of 5.1 years after RT completion [37]. In addition, the second cancer risk between IMRT and 3D-CRT was not different among all the primary tumor types with the sole exception of primary tumors of the head and neck that showed a decreased risk with IMRT.

## 4. New Insights

### 4.1. RBE and LET Optimization and Robustness Improvement

Recently, there has been increased attention on the adverse effects of PT. This analysis involves comparing the toxicity rates observed in conventional X-ray RT to determine if unexpected tissue damage could be due to an underestimated increase in RBE at the end of the proton beam range [38].

Ongoing discussions and studies are examining statistical correlations between increased side effects and variables such as RBE, employing different models and linear energy transfer (LET) distributions, but at this point in the discussion, the results remain controversial. In 2019, the American Association of Physicists in Medicine AAPM Task Group 265 (AAPM-TG265) concluded that endorsing the adoption of a variable RBE model for clinical application was premature; instead, they advocated for continuing the use of a constant RBE value of 1.1 in clinical practice [39]. Additionally, the task group highlighted a potential exception: when the end of range falls within a critical structure with a known low α/β ratio, alternative dose constraints relative to photons could be established, possibly necessitating an increase in the RBE to 1.2 or 1.3.

Niemerko et al. stated that a clear correlation between areas of toxicity and proton LET has not been found, hence suggesting that individual patient radiosensitivity plays the most significant role in terms of toxicity [40]. Conversely, studies focusing on patients with HNC have reported a robust correlation between higher LET and RBE and toxicity in the oral cavity and oropharynx [41]. Wagenaar et al. found that the sample size necessary to independently correlate the mean D·LETd of OARs with patient toxicity was prohibitively large for all considered toxicities; even with 10,000 patients, the statistical power remained below 10% for toxicity outcomes such as xerostomia, dysphagia, and the need for tube feeding [42].

**Figure 1:**
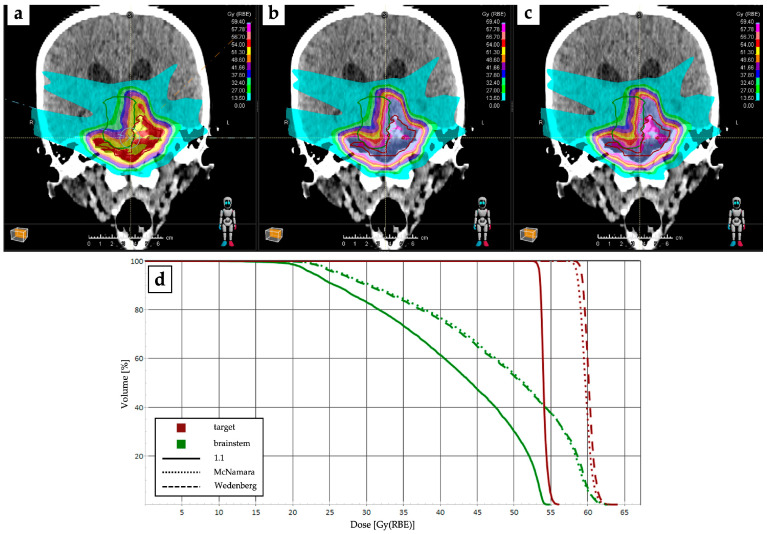
Dose distributions of a proton therapy plan (prescription dose of 54 Gy (RBE)) optimized with RBE = 1.1 (**a**) and recalculated with McNamara (**b**) and Wedenberg (**c**) RBE models, respectively. Relative dose–volume histograms (DVHs) for target (dark red) and brainstem (green) are reported in (**d**).

The latest upgrades in commercial Proton Therapy Treatment Planning Systems (PT TPSs) have introduced LET-based objectives to reduce the maximum LET of particles delivered above a certain threshold dose [43]. While the clinical impact of minimizing the contribution of high-LET particles to OARs has yet to be demonstrated, integrating dose- and LET-based objectives into the optimization process could potentially lead to superior treatment plans compared to classical robustness approaches, which only consider setup and range uncertainties. Figure 1 provides an example of PT plan changes after implementation with RBE models.

Among the potential new approaches and methodologies for treating HNC with protons, it is worth mentioning the use of transmission beams (TBs) [44,45]. Unlike standard proton therapy (IMPT) where the Bragg peak is intentionally positioned within the target, in the case of the TB technique, the plateau of a single high beam energy is used to hit the target. Since the beam passes through the patient, delivering the dose in the plateau region, an increase or decrease in the range does not significantly alter the dose deposited by the beam itself. This type of dose delivery technique therefore generates particularly robust treatment plans, as they are insensitive to range uncertainties but only to intrinsic (2D) uncertainties due to setup errors.

This could be particularly promising in the context of head and neck PT, for which dose range uncertainties due the filling/emptying of cavities or the presence of metallic implants and artifacts are very limiting, often requiring subsequent plan re-calculation for the re-evaluation computed tomography (CT) scans acquired during treatment.

Moreover, given that the highest beam energies are typically delivered at high dose rates, particularly for cyclotrons where the highest beam energy is often rapidly degraded by passive elements inserted along the beam path, high-energy TB plans can be delivered at ultra-high dose rates (UHDRs), hence in FLASH modality [46]. This contrasts the current Bragg peak plans, which need energy modulation, thereby reducing beam intensity while increasing beam time.

Lastly, TB technique can be adopted both for gantry geometries and in sitting position if a treatment chair is available. However, as a counterbalance, the TB technique requires the use of multiple treatment fields (at least 7 and up to 10), thus increasing the low dose bath, although the comparison remains favorable with respect to more advanced photon arc techniques.

### 4.2. Multi-Ion Radiotherapy and Proton Minibeam Radiation Therapy

Although painting the LET in a tumor represents the most immediate solution to focus the therapeutic dose on the most radioresistant areas, this approach faces some intrinsic limitations attributable to the physical properties of the beams themselves. To overcome this issue, with a view to increase patient-specific treatments, multi-ion radiotherapy (MIRT) has been developed.

This technique, still in the experimental phase and requiring significant technical development in particle accelerators, would allow the use of various ion species in addition to protons (e.g., carbon, helium, oxygen, neon) by exploiting their specific LET and, therefore, RBE for specific areas of the target. For example, lower-LET beams such as helium may offer improved dosage margins where tumors are close to normoxic, healthy tissues, while irradiation with higher-LET ion species can be stratified into hypoxic and radioresistant regions [47]. MIRT, for now in a utopian vision of clinical practice, could find application in the treatment of NPC both in the adjuvant and reirradiation setting together with HNC with intrinsic radioresistance.

Promising results have also been recently reported by combining the advantages of proton dose deposition pattern with the use of submillimetric beams, as previously introduced with X-rays in the so-called spatially fractionated radiotherapy (SFRT) [48,49]. Proton minibeam radiation therapy (pMBRT) has demonstrated to improve normal tissue sparing [50] while providing comparable or superior tumor control compared to standard PT in preclinical settings [51].

Significant steps towards the implementation of an adequate plan optimization workflow with this technique were recently described [52,53], but further research is needed to unveil the hidden potential of pMBRT in different oncological settings, including HNC.

### 4.3. Favorable Biological Properties and Immunogenic Effects

The exact mechanisms underlying the biologically different effects elicited by protons with respect to photons have not yet been fully clarified. Although the generic proton RBE is commonly accepted to be equal to 1.1, proton beams exhibit higher LET with increasing depth in the Spread Out Bragg Peak (SOBP), which leads to biological advantages compared to X-rays [54,55,56].

Experiments on HNSCC cell lines allowed to prove that mRNA levels of genes involved in angiogenesis, inflammation, proliferation, and anti-tumor immunity are significantly lower after PT; indeed, while both proton and photon irradiation are known to increase the vascular endothelial growth factor C (VEGF-C), a key factor linked to the metastatic dissemination of cancer cells and unfavorable prognosis, and proton exposure results in significantly reduced levels [57].

HNSCC cells subjected to PT or photon-based RT show different protein expression profiles too. In detail, proteins involved in DNA damage repair, cell cycle progression, and survival are downregulated after proton exposure especially in HPV-positive cells, evidence that is detectable even at different time points [58]. Moreover, both proton and carbon ion RTs have been shown to suppress in vitro tumor cell migration in a dose-dependent manner through the inhibition of collagen degradation by matrix metalloproteinases (MMP) [59]. However, further studies are needed to better understand the clinical impact of these findings.

The concept that HNSCC cells exhibit increased sensitivity to PT is rapidly spreading, but the underlying mechanisms still deserve deeper investigation. Wang et al. were the first to study two HPV-positive and two HPV-negative human HNSCC cell lines after a single dose of 4 Gy of photon RT and PT; PT was found to lead to a greater number of unrepaired double-stranded breaks at 24 h, causing higher levels of mitotic catastrophe than photon RT [60]. These potential disparities in cell death mechanisms, if confirmed in vivo, could pave the way for the exploration of therapeutic approaches involving targeted therapies that interfere with different cell death pathways.

With regard to the synergistic benefits of combining immunotherapy and RT, the advent of PT has further boosted the interest towards this multimodal approach. The unique physical properties of protons with their sharp dose fall-off allow to spare much more normal tissue and reduce the exposure of circulating T-lymphocytes and other radiosensitive immune cells, resulting in a less immune-suppressive effect than photons [61]. Although lymphocyte sparing has not yet been shown to correlate with an enhanced immune response, reduced radiation-induced lymphopenia is a known independent positive prognostic factor for patients with HNC [62]. Multiple elements of the tumor microenvironment also contribute to mechanisms to evade immune surveillance, including small tumor-derived extracellular vesicles, also known as exosomes [63,64]. A recent in silico paper by Chimote et al. on primary HNSCC cells irradiated with 5 Gy PT or X-ray RT reported that PT lead to the production of 75% fewer exosomes compared to X-ray RT and non-irradiated HNSCC cells [65]. The inhibition of exosome production could highlight the advantages of PT over photon-based RT in enhancing anti-tumor immunity, as well as the potential benefits of combining PT with immune checkpoint inhibitors and various other forms of immunotherapy.

Apart from the aforementioned physical superiority, the increase in proton LET at or around the Bragg peak can potentially justify the activation of different DNA damage pathways that trigger immune-stimulatory effects. Indeed, protons can cause significantly complex double-stranded DNA damage than photons, and its extrusion from the nucleus to the cytosol makes it act as a radio-induced antigen capable of activating the transcription of type I interferon genes and consequently the recruitment of dendritic cells and cytotoxic T-lymphocytes (CTLs) [66]. Moreover, PT can promote interactions between CTLs and tumor cells by stimulating increased calreticulin expression and its translocation to the cell surface, even in those typically resistant cells such as cancer stem cells [67].

Even if HNSCC cell lines showed a different expression of Programmed Death Ligand-1 (PD-L1) and Cytotoxic T-lymphocyte Antigen 4 (CTLA4) after exposure to protons with respect to X-rays [58], additional investigations are mandatory to unveil the potential benefits of combining different immune checkpoint inhibitors with PT, which are needed to unveil the hidden potential of pMBRT in different oncological settings, including HNC.

### 4.4. De-Escalation Strategy

Specifically considering protons’ physical properties, the extreme conformity achieved with PT can facilitate volume de-escalation approaches, particularly in patients with HPV-associated OPC and NPC, as it eliminates biases related to low and intermediate doses.

Traditionally, HPV-associated OPC was primarily managed through RT, administered as IMRT and concurrent chemotherapy, but recent clinical trials have explored various strategies for less aggressive treatment approaches [68]. One of them is represented by the application of minimally invasive surgery, such as TORS.

Due to the proximity of the highest nodal station to the oropharyngeal mucosa, the omission of low-to-intermediate doses, impossible with highly conformal radiotherapy (i.e., IMRT), could provide valuable insights into the efficacy of not irradiating the primary tumor bed after TORS. Long-term data on overall survival and progression-free survival of primary photon RT compared to primary transoral surgery from the ORATOR2 randomized phase 2 clinical trial are still preliminary [69]. Meanwhile, an ongoing observational study at the M.D. Anderson Cancer Center (NCT02663583) is investigating objective functional outcomes of low-risk OPC receiving IMPT or TORS [70].

Unilateral neck irradiation is another de-escalation strategy that can reduce toxicity and improve QoL for selected patients with early-stage and lateralized OPC [71,72,73], and further unintended dose reduction in the contralateral neck can be achieved by taking advantage of the steep dose gradient of protons [74,75]. Recently, a multi-institutional prospective analysis conducted by the Proton Collaborative Group on patients with OPC subjected to ipsilateral PT reported an excellent contralateral neck failure rate, comparable to failure rates observed with photon RT [76].

Preclinical studies reported that the clonogenic survival after PT was lower in the HPV-associated cell lines with respect to HPV-negative cell lines, suggesting that HPV-associated OPC may be more sensitive to PT [60]. This is the rationale behind an ongoing phase 2 study led by the Memorial Sloan Kettering Cancer Center in which selected low-grade non-hypoxic HPV-associated OPCs receive de-escalated RT of 30 Gy over three weeks including PT concurrent with two cycles of standard chemotherapy (NCT03323463) [70]; the aim is to demonstrate that locoregional control at two years for this patient cohort is not inferior to the current standard of care.

An innovative approach to dose de-escalation was recently proposed by the ongoing HYpofractionated Dose-redistributed RAdiotherapy (HYDRA) trial (NCT05364411) [70]. First, considering that higher doses per fraction on macroscopic tumor are correlated with increased immunogenicity, 20 fractions instead of the conventional 35 fractions are proposed for all HNSCCs amenable to definitive RT with or without a concomitant radiosensitizer. Then, a radiation dose redistribution is performed toward a higher dose within the tumor center and a lower dose outside the target volume, which can result in a significant reduction in radiation-induced lymphodepletion. Finally, PT is used to discover its additional effect on antitumor immunity compared to photon hypofractionation and conventional fractionated RT.

Focusing on NPC, although characterized by a high incidence of cervical lymph node metastases, usually nodal involvement follow an ordered pattern, with retropharyngeal and level II lymph nodes being the most commonly involved, followed by levels III, V, and IV [77]. With these premises, interest is growing in de-intensification strategies that reduce RT volumes and treatment-related side effects, especially in N0–N1 patients; to date, no significant differences have emerged in overall survival, disease-free survival, and locoregional control by reducing or omitting irradiation of the lower neck with prophylactic intent [78,79,80,81], but further evidence is needed to confirm these results. Furthermore, whether IMPT and its dosimetric advantage over IMRT can translate into worse local control, especially in the contralateral lower neck-sparing approach, is still a question open for discussion [82].

## 5. Limitations and Future Directions

The substantial obstacle impending the widespread adoption of PT lies in the considerable upfront costs involved in building and operating proton beam therapy facilities. The elevated treatment expenses are coupled with a lack of conclusive high-level evidence and consequently with reimbursement challenges [83]. Indeed, while PT exhibits superior dose distribution characteristics compared to photon RT, there is still a need for quantifiable clinical evidence to establish its clear advantage; only a thorough assessment of proton effectiveness may warrant a reconsideration of government health policies and insurance coverage. To date, most of the available evidence comes from retrospective studies, such as the largest and most recent cohort study by Chang et al. which is the first to compare the oncologic outcomes of PT and IMRT for patients with HNSCC [84]. Using a propensity score matching approach, PT was shown to be associated with improved overall survival and cancer-specific survival and reduced locoregional recurrence rates, but randomized clinical trials and prospective cohort studies are mandatory to confirm these findings.

With regard to the cost-effectiveness of protons, it remains controversial even if several analyses have been conducted in recent years. In the systematic review by Verma et al., PT was not the most economical option for all cancer types, but it proved to be cost-effective in selected patients with HNC at higher risk of acute mucosal toxicities [85]. More recently, Sher et al. performed a cost-effectiveness analysis of IMPT versus IMRT in node-positive oropharynx cancer investigating both the payer and societal perspectives [86]; they found that IMPT had the potential to be a cost-effective therapy in the payer perspective only for younger patients positive for HPV experiencing a halving of both xerostomia and gastrostomy use, further supporting the hypothesis that only certain subgroups of patients could benefit economically from protons [87].

It has been estimated that, for Chinese patients with NPC, IMPT should yield a reduction in normal tissue complication probability (NTCP) of at least 17–39% depending on patient’s age in order to be considered cost-effective [88]. However, to reach exhaustive conclusions, there is a need for further reliable analysis that take into account as many salient logistical and economic variables as possible in addition to all probable treatment complications.

Unfortunately, another relevant issue revolving around PT is the large gap between the number of patients who may benefit from this kind of treatment and those who have access to it. Lee et al. extracted data of all patients with HNC subjected to RT between 2005 and 2014 from the National Cancer Database in the United States and performed a multivariable logistic regression to correlate socioeconomic factors associated with PT compared with other RT modalities [89]; interestingly, patients older than 65 years, of Hispanic ethnicity, located in the South or Midwest, or without high school education were associated with a reduced likelihood of receiving PT. In agreement with these findings, a subsequent analysis by McCall et al. highlighted that, in the American HNC setting, the most commonly reported indicators of disparity in the access to advanced RT techniques are race, age, and private insurance [90]. Focusing on ethnic inequities in insurance coverage, Black, Indigenous, and People of Color (BIPOC) received a higher proton-unfavorable rating than non-Hispanic White patients (24.9% vs. 18.4%, *p* = 0.005); in other words, PT was significantly more likely to be considered “experimental” or “medically not necessary” for their diagnosis of primary HNC [91]. Additionally, among all patients who were denied insurance approval, the median time for both decision communication and initiation of any other RT modality was significantly longer for BIPOC patients.

PT in HNC still holds immense potential for advancement and innovation. To fully exploit this promising modality, it is imperative to undertake a multifaceted approach that integrates various fields of research, collaboration, and clinical application:

- Advancements in preclinical models, both in vitro and in vivo, are essential for unveiling the underlying mechanisms of PT and its interactions with other treatment modalities. By fostering collaborations across physics, medicine, and radiobiology, we can refine our understanding of the biological effects of protons and optimize treatment strategies.
- Defining appropriate endpoints for preclinical and clinical studies is paramount for accurately assessing the effectiveness of PT. By establishing standardized endpoints, consistency across studies can be ensured and data comparison can be facilitated, ultimately driving evidence-based decision making in clinical practice.
- Enhancing the methodology and quality control measures of clinical studies is essential for ensuring the validity and reliability of research findings. By standardizing protocols, implementing rigorous quality assurance procedures, and continuously monitoring data integrity, we can strengthen the credibility of PT research and foster greater confidence in its clinical utility.
- Integrating advanced imaging techniques, omic sciences (e.g., proteomics, genomics, metabolomics, transcriptomics, and radiomics), and individual patients’ biomarkers into the real-time assessment of tumor response is crucial for optimizing PT delivery and detecting resistance patterns. By leveraging these tools, we can monitor treatment response more accurately, enabling timely adjustments to therapy and improving patient outcomes.
- Establishing networks between hadrontherapy centers regionally, nationally, and internationally facilitates the exchange of knowledge, resources, and best practices. Through collaborative efforts, centers can streamline processes, share expertise, and collectively address the challenge of improving patient care.
- Leveraging the existing hadrontherapy facilities to launch larger multinational trials targeting common cancers, including HNC. By pooling resources and collaborating, we can conduct trials with sufficient statistical power to assess the effectiveness of PT.
- Collaborating with the pharmaceutical industry is crucial for identifying and prioritizing new combinations of PT with emerging therapeutic agents. By fostering partnerships, we can accelerate the development of novel treatment regimens that enhance the efficacy of protons while minimizing adverse effects.

## 6. Conclusions

PT has emerged as a promising treatment modality in the management of HNC with growing evidence supporting its dose-sparing effect and clinical implications in reducing normal tissue toxicity while maintaining or even improving treatment efficacy. Furthermore, emerging evidence suggests that PT may modulate the tumor microenvironment in ways that enhance antitumor immune responses potentially synergizing with immunotherapeutic agents. Finally, by leveraging RBE and LET optimization, robustness improvement, and cutting-edge technologies such as MIRT and pMBRT, clinicians can explore several innovative ways to fully exploit protons in the fight against HNC.

By prioritizing research collaboration and implementing evidence-based practice, PT could be integrated into standard treatment algorithms, thereby redefining the therapeutic landscape of HNC in the years to come.

## Figures and Tables

**Table 1:** Ongoing clinical trials investigating photon radiotherapy versus proton radiotherapy in head and neck carcinoma.

Acronym/ClinicalTrials.gov ID	Country	Study Population	Intervention	Primary Endpoints	Secondary Endpoints	Study Type	Study Start
NCT01893307	United States	Stage III–IVB oropharyngeal squamous cell carcinoma (AJCC v7)	IMRT vs. IMPT with concurrent chemotherapy	-Grade 3–5 late toxicity (CTCAE v4.0) -3-year PFS	Disease-related and patient-reported outcomes (including QALY and cost–benefit economic analysis)	Randomized phase II/III trial	August 2013
NCT02923570	United States	HNSCC requiring ipsilateral radiation, salivary gland cancer, skin cancer, and melanoma	Standard dose of 60–66 Gy of IMRT vs. PT	Grade ≥ 2 acute mucositis (CTCAE v4.0)	n.a.	Randomized phase II trial	October 2016
ARTSCAN V/NCT03829033	Sweden	Early squamous cell carcinoma of the tonsil	Photon RT vs. PT	Acute and late toxicity (CTCAE v4.0)	n.a.	Randomized phase II trial	January 2019
NCT04528394	China	HNSCC (nasopharynx)	Photon RT combined with CIRT vs. PT combined with CIRT<	Grade ≥ 2 xerostomia (CTCAE v4.03)	-OS, PFS, LRC -acute and late toxicities (CTCAE v4.03)	Randomized phase II trial	April 2019
TORPEdO	United Kingdom	HNSCC (locally advanced oropharynx)	70 Gy/56 Gy in 33 fractions using an SIB technique of IMRT vs. IMPT with concurrent chemotherapy	-UW-QoL v4.0 -gastrostomy dependence or grade 3 weight loss (CTCAE v5.0)	-validate a NTCP model -pattern of health-related quality of life -tube feeding status -weight loss >10% from baseline -acute and late toxicity (CTCAE v5.0) -clinician-rated swallowing function assessment -PSS-HN -LRC, OS -cost-effectiveness	Phase III, multicenter, open-label, randomized controlled trial	February 2020
DAHANCA 35/NCT04607694	Denmark	HNSCC (pharynx or larynx)	66–68 Gy in 33–34 fractions of photon RT vs. PT with concurrent chemotherapy	-Grade ≥ 2 late dysphagia (DAHANCA score) -Grade ≥ 4 xerostomia (EORTC QLQ-HN35)	-LRC, OS, DFS, DSS -acute and late toxicity -EORTC QLQ-C30, MD Anderson Dysphagia Index, EQ-5D	Two parallel randomized studies	October 2020
PRO-IMMUNO/NCT06016699	Netherlands	HNSCC	Photon RT vs. PT with concurrent chemotherapy	Difference in antigen-specific immunity	-Differences in composition and function of circulating immune cells -Immune infiltrate composition within the primary tumor tissue	Observational	September 2021
HYDRA/NCT05364411	Netherlands	HNSCC	Mean dose of 59 Gy in 20 fractions of photon RT vs. PT	Grade 3–4 late toxicity (CTCAE v5.0)	-Objective response (RECIST v1.1) -In-field and nodal elective field tumor control -Immune profile	Two parallel non-comparative phase-I trials	October 2022

Abbreviations: AJCC v7, American Joint Committee on Cancer version 7; IMRT, intensity-modulated photon therapy; IMPT, intensity-modulated proton therapy; CTCAE, Common Terminology Criteria for Adverse Events; PFS, progression-free survival; QALY, quality-adjusted life years; HNSCC, head and neck squamous cell carcinoma; PT, proton therapy; RT, radiotherapy; CIRT, carbon ion radiotherapy; OS, overall survival; LRC, locoregional control; SIB, simultaneous integrated boost; UW-QoL v4.0, University of Washington Quality of Life Questionnaire version 4; NTCP, normal tissue complication probability; PSS-HN, Performance Status Scale for Head and Neck Cancer; DAHANCA, Danish Head and Neck Cancer Group; EORTC QLQ-HN35, European Organization for Research and Treatment of Cancer Quality of Life Questionnaire Head and Neck Module; DFS, disease-free survival; DSS, disease-specific survival; EORTC QLQ-C30, European Organization for Research and Treatment of Cancer Quality of Life Questionnaire Core Module; EQ-5D, EuroQol Five Dimension Scale; RECIST, Response Evaluation Criteria in Solid Tumors; and n.a., not available.

## References

[1] Machiels J.-P., René Leemans C., Golusinski W., Grau C., Licitra L., Gregoire V., EHNS Executive Board, ESMO Guidelines Committee, ESTRO Executive Board (2020). Squamous Cell Carcinoma of the Oral Cavity, Larynx, Oropharynx and Hypopharynx: EHNS-ESMO-ESTRO Clinical Practice Guidelines for Diagnosis, Treatment and Follow-Up. Ann. Oncol. Off. J. Eur. Soc. Med. Oncol..

[2] Bossi P., Chan A.T., Licitra L., Trama A., Orlandi E., Hui E.P., Halámková J., Mattheis S., Baujat B., Hardillo J. (2021). Nasopharyngeal Carcinoma: ESMO-EURACAN Clinical Practice Guidelines for Diagnosis, Treatment and Follow-Up. Ann. Oncol..

[3] Nutting C.M., Morden J.P., Harrington K.J., Urbano T.G., Bhide S.A., Clark C., Miles E.A., Miah A.B., Newbold K., Tanay M. (2011). Parotid-Sparing Intensity Modulated versus Conventional Radiotherapy in Head and Neck Cancer (PARSPORT): A Phase 3 Multicentre Randomised Controlled Trial. Lancet Oncol..

[4] Peng G., Wang T., Yang K., Zhang S., Zhang T., Li Q., Han J., Wu G. (2012). A Prospective, Randomized Study Comparing Outcomes and Toxicities of Intensity-Modulated Radiotherapy vs. Conventional Two-Dimensional Radiotherapy for the Treatment of Nasopharyngeal Carcinoma. Radiother. Oncol..

[5] AIOM-Registri Tumori Italiani-SIAPEC-PASSI-PASSI D’ARGENTO-ONS-Fondazione AIOM I Numeri Del Cancro in Italia 2022. https://www.aiom.it/wp-content/uploads/2022/12/2022_AIOM_NDC-web.pdf.

[6] Siegel R.L., Miller K.D., Fuchs H.E., Jemal A. (2022). Cancer Statistics, 2022. CA Cancer J. Clin..

[7] Ringash J. (2015). Survivorship and Quality of Life in Head and Neck Cancer. J. Clin. Oncol..

[8] De Felice F., Locati L.D., Ronchi S., Thariat J., Orlandi E. (2022). Quality of Life and Financial Toxicity after (Chemo)Radiation Therapy in Head and Neck Cancer: Are There Any Sex- or Gender-Related Differences?. Tumori J..

[9] McDowell L., Corry J., Ringash J., Rischin D. (2020). Quality of Life, Toxicity and Unmet Needs in Nasopharyngeal Cancer Survivors. Front. Oncol..

[10] Beddok A., Vela A., Calugaru V., Tessonnier T., Kubes J., Dutheil P., Gerard A., Vidal M., Goudjil F., Florescu C. (2020). Proton Therapy for Head and Neck Squamous Cell Carcinomas: A Review of the Physical and Clinical Challenges. Radiother. Oncol..

[11] Tambas M., Van Der Laan H.P., Steenbakkers R.J.H.M., Doyen J., Timmermann B., Orlandi E., Hoyer M., Haustermans K., Georg P., Burnet N.G. (2022). Current Practice in Proton Therapy Delivery in Adult Cancer Patients across Europe. Radiother. Oncol..

[12] Gaikwad U., Bajpai J., Jalali R. (2023). Combinatorial Approach of Immuno-proton Therapy in Cancer: Rationale and Potential Impact. Asia Pac. J. Clin. Oncol..

[13] Lee A., Kitpanit S., Chilov M., Langendijk J.A., Lu J., Lee N.Y. (2021). A Systematic Review of Proton Therapy for the Management of Nasopharyngeal Cancer. Int. J. Part. Ther..

[14] Li X., Kitpanit S., Lee A., Mah D., Sine K., Sherman E.J., Dunn L.A., Michel L.S., Fetten J., Zakeri K. (2021). Toxicity Profiles and Survival Outcomes Among Patients With Nonmetastatic Nasopharyngeal Carcinoma Treated With Intensity-Modulated Proton Therapy vs Intensity-Modulated Radiation Therapy. JAMA Netw. Open.

[15] Wu P.-W., Huang C.-C., Lee Y.-S., Chou Y.-C., Fan K.-H., Lin C.-Y., Huang B.-S., Yang S.-W., Huang C.-C., Chang P.-H. (2022). Post-Irradiation Sinus Mucosa Disease in Nasopharyngeal Carcinoma Patients Treated with Intensity-Modulated Proton Therapy. Cancers.

[16] Holliday E.B., Garden A.S., Rosenthal D.I., Fuller C.D., Morrison W.H., Gunn G.B., Phan J., Beadle B.M., Zhu X.R., Zhang X. (2015). Proton Therapy Reduces Treatment-Related Toxicities for Patients with Nasopharyngeal Cancer: A Case-Match Control Study of Intensity-Modulated Proton Therapy and Intensity-Modulated Photon Therapy. Int. J. Part. Ther..

[17] McDonald M.W., Liu Y., Moore M.G., Johnstone P.A.S. (2016). Acute Toxicity in Comprehensive Head and Neck Radiation for Nasopharynx and Paranasal Sinus Cancers: Cohort Comparison of 3D Conformal Proton Therapy and Intensity Modulated Radiation Therapy. Radiat. Oncol..

[18] Chou Y.-C., Fan K.-H., Lin C.-Y., Hung T.-M., Huang B.-S., Chang K.-P., Kang C.-J., Huang S.-F., Chang P.-H., Hsu C.-L. (2021). Intensity Modulated Proton Beam Therapy versus Volumetric Modulated Arc Therapy for Patients with Nasopharyngeal Cancer: A Propensity Score-Matched Study. Cancers.

[19] Williams V.M., Parvathaneni U., Laramore G.E., Aljabab S., Wong T.P., Liao J.J. (2021). Intensity-Modulated Proton Therapy for Nasopharynx Cancer: 2-Year Outcomes from a Single Institution. Int. J. Part. Ther..

[20] Liu C.-H., Lin C.-Y., Huang B.-S., Wei Y.-C., Chang T.-Y., Yeh C.-H., Sung P.-S., Jiang J.-L., Lin L.-Y., Chang J.T.-C. (2023). Risk of Temporal Lobe Necrosis between Proton Beam and Volumetric Modulated Arc Therapies in Patients with Different Head and Neck Cancers. Radiat. Oncol..

[21] Cozzi L., Fogliata A., Lomax A., Bolsi A. (2001). A Treatment Planning Comparison of 3D Conformal Therapy, Intensity Modulated Photon Therapy and Proton Therapy for Treatment of Advanced Head and Neck Tumours. Radiother. Oncol..

[22] Van De Water T.A., Lomax A.J., Bijl H.P., De Jong M.E., Schilstra C., Hug E.B., Langendijk J.A. (2011). Potential Benefits of Scanned Intensity-Modulated Proton Therapy Versus Advanced Photon Therapy With Regard to Sparing of the Salivary Glands in Oropharyngeal Cancer. Int. J. Radiat. Oncol..

[23] Van De Water T.A., Lomax A.J., Bijl H.P., Schilstra C., Hug E.B., Langendijk J.A. (2012). Using a Reduced Spot Size for Intensity-Modulated Proton Therapy Potentially Improves Salivary Gland-Sparing in Oropharyngeal Cancer. Int. J. Radiat. Oncol..

[24] Bagley A.F., Ye R., Garden A.S., Gunn G.B., Rosenthal D.I., Fuller C.D., Morrison W.H., Phan J., Sturgis E.M., Ferrarotto R. (2020). Xerostomia-Related Quality of Life for Patients with Oropharyngeal Carcinoma Treated with Proton Therapy. Radiother. Oncol..

[25] Aggarwal P., Hutcheson K.A., Garden A.S., Mott F.E., Lu C., Goepfert R.P., Fuller C.D., Lai S.Y., Gunn G.B., Chambers M.S. (2021). Determinants of Patient-reported Xerostomia among Long-term Oropharyngeal Cancer Survivors. Cancer.

[26] Cao J., Zhang X., Jiang B., Chen J., Wang X., Wang L., Sahoo N., Zhu X.R., Ye R., Blanchard P. (2021). Intensity-Modulated Proton Therapy for Oropharyngeal Cancer Reduces Rates of Late Xerostomia. Radiother. Oncol..

[27] Blanchard P., Garden A.S., Gunn G.B., Rosenthal D.I., Morrison W.H., Hernandez M., Crutison J., Lee J.J., Ye R., Fuller C.D. (2016). Intensity-Modulated Proton Beam Therapy (IMPT) versus Intensity-Modulated Photon Therapy (IMRT) for Patients with Oropharynx Cancer—A Case Matched Analysis. Radiother. Oncol..

[28] Manzar G.S., Lester S.C., Routman D.M., Harmsen W.S., Petersen M.M., Sloan J.A., Mundy D.W., Hunzeker A.E., Amundson A.C., Anderson J.L. (2020). Comparative Analysis of Acute Toxicities and Patient Reported Outcomes between Intensity-Modulated Proton Therapy (IMPT) and Volumetric Modulated Arc Therapy (VMAT) for the Treatment of Oropharyngeal Cancer. Radiother. Oncol..

[29] Zhang W., Zhang X., Yang P., Blanchard P., Garden A.S., Gunn B., Fuller C.D., Chambers M., Hutcheson K.A., Ye R. (2017). Intensity-Modulated Proton Therapy and Osteoradionecrosis in Oropharyngeal Cancer. Radiother. Oncol..

[30] Singh A., Kitpanit S., Neal B., Yorke E., White C., Yom S.K., Randazzo J.D., Wong R.J., Huryn J.M., Tsai C.J. (2023). Osteoradionecrosis of the Jaw Following Proton Radiation Therapy for Patients With Head and Neck Cancer. JAMA Otolaryngol. Neck Surg..

[31] Yahya N., Manan H.A. (2023). Quality of Life and Patient-Reported Outcomes Following Proton Therapy for Oropharyngeal Carcinoma: A Systematic Review. Cancers.

[32] Sio T.T., Lin H.-K., Shi Q., Gunn G.B., Cleeland C.S., Lee J.J., Hernandez M., Blanchard P., Thaker N.G., Phan J. (2016). Intensity Modulated Proton Therapy Versus Intensity Modulated Photon Radiation Therapy for Oropharyngeal Cancer: First Comparative Results of Patient-Reported Outcomes. Int. J. Radiat. Oncol..

[33] Sharma S., Zhou O., Thompson R., Gabriel P., Chalian A., Rassekh C., Weinstein G.S., O’Malley B.W., Aggarwal C., Bauml J. (2018). Quality of Life of Postoperative Photon versus Proton Radiation Therapy for Oropharynx Cancer. Int. J. Part. Ther..

[34] Smith G.L., Fu S., Ning M.S., Nguyen D.-K., Busse P.M., Foote R.L., Garden A.S., Gunn G.B., Fuller C.D., Morrison W.H. (2021). Work Outcomes after Intensity-Modulated Proton Therapy (IMPT) versus Intensity-Modulated Photon Therapy (IMRT) for Oropharyngeal Cancer. Int. J. Part. Ther..

[35] Ng S.P., Pollard C., Kamal M., Ayoub Z., Garden A.S., Bahig H., Gunn G.B., Frank S.J., Skinner H.D., Phan J. (2019). Risk of Second Primary Malignancies in Head and Neck Cancer Patients Treated with Definitive Radiotherapy. NPJ Precis. Oncol..

[36] Jain V., Irmen P., O’Reilly S., Vogel J.H., Lin L., Lin A. (2020). Predicted Secondary Malignancies Following Proton versus Photon Radiation for Oropharyngeal Cancers. Int. J. Part. Ther..

[37] Xiang M., Chang D.T., Pollom E.L. (2020). Second Cancer Risk after Primary Cancer Treatment with Three-Dimensional Conformal, Intensity-Modulated, or Proton Beam Radiation Therapy. Cancer.

[38] Underwood T.S.A., McNamara A.L., Appelt A., Haviland J.S., Sørensen B.S., Troost E.G.C. (2022). A Systematic Review of Clinical Studies on Variable Proton Relative Biological Effectiveness (RBE). Radiother. Oncol..

[39] Paganetti H., Blakely E., Carabe-Fernandez A., Carlson D.J., Das I.J., Dong L., Grosshans D., Held K.D., Mohan R., Moiseenko V. (2019). Report of the AAPM TG-256 on the Relative Biological Effectiveness of Proton Beams in Radiation Therapy. Med. Phys..

[40] Niemierko A., Schuemann J., Niyazi M., Giantsoudi D., Maquilan G., Shih H.A., Paganetti H. (2021). Brain Necrosis in Adult Patients After Proton Therapy: Is There Evidence for Dependency on Linear Energy Transfer?. Int. J. Radiat. Oncol..

[41] Fossum C.C., Beltran C.J., Whitaker T.J., Ma D.J., Foote R.L. (2017). Biological Model for Predicting Toxicity in Head and Neck Cancer Patients Receiving Proton Therapy. Int. J. Part. Ther..

[42] Wagenaar D., Schuit E., Van Der Schaaf A., Langendijk J.A., Both S. (2021). Can the Mean Linear Energy Transfer of Organs Be Directly Related to Patient Toxicities for Current Head and Neck Cancer Intensity-Modulated Proton Therapy Practice?. Radiother. Oncol..

[43] Traneus E., Ödén J. (2019). Introducing Proton Track-End Objectives in Intensity Modulated Proton Therapy Optimization to Reduce Linear Energy Transfer and Relative Biological Effectiveness in Critical Structures. Int. J. Radiat. Oncol..

[44] Mou B., Beltran C.J., Park S.S., Olivier K.R., Furutani K.M. (2014). Feasibility of Proton Transmission-Beam Stereotactic Ablative Radiotherapy versus Photon Stereotactic Ablative Radiotherapy for Lung Tumors: A Dosimetric and Feasibility Study. PLoS ONE.

[45] Van Marlen P., Dahele M., Folkerts M., Abel E., Slotman B.J., Verbakel W. (2021). Ultra-High Dose Rate Transmission Beam Proton Therapy for Conventionally Fractionated Head and Neck Cancer: Treatment Planning and Dose Rate Distributions. Cancers.

[46] Van Marlen P., Dahele M., Folkerts M., Abel E., Slotman B.J., Verbakel W.F.A.R. (2020). Bringing FLASH to the Clinic: Treatment Planning Considerations for Ultrahigh Dose-Rate Proton Beams. Int. J. Radiat. Oncol..

[47] Ebner D.K., Frank S.J., Inaniwa T., Yamada S., Shirai T. (2021). The Emerging Potential of Multi-Ion Radiotherapy. Front. Oncol..

[48] Ortiz R., De Marzi L., Prezado Y. (2022). Preclinical Dosimetry in Proton Minibeam Radiation Therapy: Robustness Analysis and Guidelines. Med. Phys..

[49] Reaz F., Sitarz M.K., Traneus E., Bassler N. (2023). Parameters for Proton Minibeam Radiotherapy Using a Clinical Scanning Beam System. Acta Oncol..

[50] Girst S., Greubel C., Reindl J., Siebenwirth C., Zlobinskaya O., Walsh D.W.M., Ilicic K., Aichler M., Walch A., Wilkens J.J. (2016). Proton Minibeam Radiation Therapy Reduces Side Effects in an In Vivo Mouse Ear Model. Int. J. Radiat. Oncol..

[51] Prezado Y., Jouvion G., Guardiola C., Gonzalez W., Juchaux M., Bergs J., Nauraye C., Labiod D., De Marzi L., Pouzoulet F. (2019). Tumor Control in RG2 Glioma-Bearing Rats: A Comparison Between Proton Minibeam Therapy and Standard Proton Therapy. Int. J. Radiat. Oncol..

[52] Reaz F., Traneus E., Bassler N. (2024). Tuning Spatially Fractionated Radiotherapy Dose Profiles Using the Moiré Effect. Sci. Rep..

[53] Ortiz R., Belshi R., De Marzi L., Prezado Y. (2023). Proton Minibeam Radiation Therapy for Treating Metastases: A Treatment Plan Study. Med. Phys..

[54] Paganetti H., Niemierko A., Ancukiewicz M., Gerweck L.E., Goitein M., Loeffler J.S., Suit H.D. (2002). Relative Biological Effectiveness (RBE) Values for Proton Beam Therapy. Int. J. Radiat. Oncol. Biol. Phys..

[55] Grassberger C., Trofimov A., Lomax A., Paganetti H. (2011). Variations in Linear Energy Transfer Within Clinical Proton Therapy Fields and the Potential for Biological Treatment Planning. Int. J. Radiat. Oncol..

[56] Paganetti H. (2014). Relative Biological Effectiveness (RBE) Values for Proton Beam Therapy. Variations as a Function of Biological Endpoint, Dose, and Linear Energy Transfer. Phys. Med. Biol..

[57] Lupu-Plesu M., Claren A., Martial S., N’Diaye P.-D., Lebrigand K., Pons N., Ambrosetti D., Peyrottes I., Feuillade J., Hérault J. (2017). Effects of Proton versus Photon Irradiation on (Lymph)Angiogenic, Inflammatory, Proliferative and Anti-Tumor Immune Responses in Head and Neck Squamous Cell Carcinoma. Oncogenesis.

[58] Wang L., Yang L., Han S., Zhu J., Li Y., Wang Z., Fan Y., Lin E., Zhang R., Sahoo N. (2020). Patterns of Protein Expression in Human Head and Neck Cancer Cell Lines Differ after Proton vs Photon Radiotherapy. Head Neck.

[59] Ogata T., Teshima T., Kagawa K., Hishikawa Y., Takahashi Y., Kawaguchi A., Suzumoto Y., Nojima K., Furusawa Y., Matsuura N. (2005). Particle Irradiation Suppresses Metastatic Potential of Cancer Cells. Cancer Res..

[60] Wang L., Han S., Zhu J., Wang X., Li Y., Wang Z., Lin E., Wang X., Molkentine D.P., Blanchard P. (2019). Proton versus Photon Radiation–Induced Cell Death in Head and Neck Cancer Cells. Head Neck.

[61] Fang P., Shiraishi Y., Verma V., Jiang W., Song J., Hobbs B.P., Lin S.H. (2017). Lymphocyte-Sparing Effect of Proton Therapy in Patients with Esophageal Cancer Treated with Definitive Chemoradiation. Int. J. Part. Ther..

[62] Liu L.-T., Chen Q.-Y., Tang L.-Q., Guo S.-S., Guo L., Mo H.-Y., Chen M.-Y., Zhao C., Guo X., Qian C.-N. (2018). The Prognostic Value of Treatment-Related Lymphopenia in Nasopharyngeal Carcinoma Patients. Cancer Res. Treat..

[63] Whiteside T.L. (2016). Exosomes and Tumor-Mediated Immune Suppression. J. Clin. Investig..

[64] Kalluri R., LeBleu V.S. (2020). The Biology, Function, and Biomedical Applications of Exosomes. Science.

[65] Chimote A.A., Lehn M.A., Bhati J., Mascia A.E., Sertorio M., Lamba M.A., Ionascu D., Tang A.L., Langevin S.M., Khodoun M.V. (2024). Proton Treatment Suppresses Exosome Production in Head and Neck Squamous Cell Carcinoma. Cancers.

[66] Durante M., Formenti S. (2020). Harnessing Radiation to Improve Immunotherapy: Better with Particles?. Br. J. Radiol..

[67] Gameiro S.R., Malamas A.S., Bernstein M.B., Tsang K.Y., Vassantachart A., Sahoo N., Tailor R., Pidikiti R., Guha C.P., Hahn S.M. (2016). Tumor Cells Surviving Exposure to Proton or Photon Radiation Share a Common Immunogenic Modulation Signature, Rendering Them More Sensitive to T Cell–Mediated Killing. Int. J. Radiat. Oncol..

[68] Orlandi E., Licitra L. (2019). The Day after De-ESCALaTE and RTOG 1016 Trials Results. Future Oncol..

[69] Palma D.A., Prisman E., Berthelet E., Tran E., Hamilton S., Wu J., Eskander A., Higgins K., Karam I., Poon I. (2022). Assessment of Toxic Effects and Survival in Treatment Deescalation With Radiotherapy vs Transoral Surgery for HPV-Associated Oropharyngeal Squamous Cell Carcinoma: The ORATOR2 Phase 2 Randomized Clinical Trial. JAMA Oncol..

[70] U.S. National Library of Medicine ClinicalTrials.Gov. https://clinicaltrials.gov.

[71] Jellema A.P., Slotman B.J., Doornaert P., Leemans C.R., Langendijk J.A. (2007). Unilateral versus Bilateral Irradiation in Squamous Cell Head and Neck Cancer in Relation to Patient-Rated Xerostomia and Sticky Saliva. Radiother. Oncol..

[72] Jensen K., Overgaard M., Grau C. (2007). Morbidity after Ipsilateral Radiotherapy for Oropharyngeal Cancer. Radiother. Oncol..

[73] Razavian N.B., D’Agostino R.B., Steber C.R., Helis C.A., Hughes R.T. (2023). Association of Unilateral Radiotherapy With Contralateral Lymph Node Failure Among Patients With Squamous Cell Carcinoma of the Tonsil: A Systematic Review and Meta-Analysis. JAMA Netw. Open.

[74] Romesser P.B., Cahlon O., Scher E., Zhou Y., Berry S.L., Rybkin A., Sine K.M., Tang S., Sherman E.J., Wong R. (2016). Proton Beam Radiation Therapy Results in Significantly Reduced Toxicity Compared with Intensity-Modulated Radiation Therapy for Head and Neck Tumors That Require Ipsilateral Radiation. Radiother. Oncol..

[75] Press R.H., Bakst R.L., Sharma S., Kabarriti R., Garg M.K., Yeh B., Gelbum D.Y., Hasan S., Choi J.I., Barker C.A. (2021). Clinical Review of Proton Therapy in the Treatment of Unilateral Head and Neck Cancers. Int. J. Part. Ther..

[76] Mumaw D.A., Hazy A.J., Vayntraub A., Quinn T.J., Salari K., Chang J.H., Kalman N., Katz S., Urbanic J., Press R.H. (2024). Low Contralateral Failure Rate with Unilateral Proton Beam Radiotherapy for Oropharyngeal Squamous Cell Carcinoma: A Multi-Institutional Prospective Study from the Proton Collaborative Group. Radiother. Oncol..

[77] Ho F.C., Tham I.W., Earnest A., Lee K.M., Lu J.J. (2012). Patterns of Regional Lymph Node Metastasis of Nasopharyngeal Carcinoma: A Meta-Analysis of Clinical Evidence. BMC Cancer.

[78] Chen M., Tang L., Sun Y., Mao Y., Li W., Guo R., Liu L., Li L., Lin A., Ma J. (2014). Treatment Outcomes and Feasibility of Partial Neck Irradiation for Patients with Nasopharyngeal Carcinoma with Only Retropharyngeal Lymph Node Metastasis after Intensity-modulated Radiotherapy. Head Neck.

[79] Hu W., Zhu G., Guan X., Wang X., Hu C. (2013). The Feasibility of Omitting Irradiation to the Contralateral Lower Neck in Stage N1 Nasopharyngeal Carcinoma Patients. Radiat. Oncol..

[80] Tang L.-L., Huang C.-L., Zhang N., Jiang W., Wu Y.-S., Huang S.H., Mao Y.-P., Liu Q., Li J.-B., Liang S.-Q. (2022). Elective Upper-Neck versus Whole-Neck Irradiation of the Uninvolved Neck in Patients with Nasopharyngeal Carcinoma: An Open-Label, Non-Inferiority, Multicentre, Randomised Phase 3 Trial. Lancet Oncol..

[81] De Felice F., Marchetti C., Serpone M., Camarda A., Vischioni B., Ingargiola R., Musio D., Orlandi E. (2023). Upper-Neck Irradiation versus Standard Whole-Neck Irradiation in Nasopharyngeal Carcinoma: A Systematic Review and Meta-Analysis. Tumori J..

[82] De Felice F., Vai A., Camarda A.M., Iacovelli N.A., Orlandi E. (2022). Lower-Neck Sparing Using Proton Therapy in Patients with Uninvolved Neck Nasopharyngeal Carcinoma: Is It Safe?. J. Clin. Med..

[83] Ning M.S., Gomez D.R., Shah A.K., Kim C.R., Palmer M.B., Thaker N.G., Grosshans D.R., Liao Z., Chapman B.V., Brooks E.D. (2019). The Insurance Approval Process for Proton Radiation Therapy: A Significant Barrier to Patient Care. Int. J. Radiat. Oncol..

[84] Chang C.-L., Lin K.-C., Chen W.-M., Shia B.-C., Wu S.-Y. (2024). Comparing the Oncologic Outcomes of Proton Therapy and Intensity-Modulated Radiation Therapy for Head and Neck Squamous Cell Carcinoma. Radiother. Oncol..

[85] Verma V., Mishra M.V., Mehta M.P. (2016). A Systematic Review of the Cost and Cost-effectiveness Studies of Proton Radiotherapy. Cancer.

[86] Sher D.J., Tishler R.B., Pham N.-L., Punglia R.S. (2018). Cost-Effectiveness Analysis of Intensity Modulated Radiation Therapy Versus Proton Therapy for Oropharyngeal Squamous Cell Carcinoma. Int. J. Radiat. Oncol..

[87] Bharathi R.P., Ms A., Kamath A. (2023). A Systematic Review of the Economic Burden of Proton Therapy in Head and Neck Cancer. Asian Pac. J. Cancer Prev..

[88] Li G., Xia Y., Huang Y., Okat D., Qiu B., Doyen J., Bondiau P., Benezery K., Gao J., Qian C. (2022). Intensity-modulated Proton Radiation Therapy as a Radical Treatment Modality for Nasopharyngeal Carcinoma in China: Cost-effectiveness Analysis. Head Neck.

[89] Lee A., Kang J., Yu Y., McBride S., Riaz N., Cohen M., Sherman E., Michel L., Lee N., Tsai C.J. (2019). Trends and Disparities of Proton Therapy Use among Patients with Head and Neck Cancer: Analysis from the National Cancer Database (2005–14). Int. J. Part. Ther..

[90] McCall N.S., Liu Y., Janopaul-Naylor J., Bates J.E., Remick J.S., Rudra S., Karam S.D., Amini A., McDonald M.W., Stokes W.A. (2022). Standard but Not Equal: Disparities in Advanced Radiotherapy Techniques for Head and Neck Cancer in the United States. Int. J. Radiat. Oncol..

[91] McDonald M.W., Bates J.E., McCall N.S., Goyal S., Liu Y., Rudra S., Remick J.S., Tian S., El-Deiry M.W., Saba N.F. (2023). Insurance Authorization and Access to Proton Therapy for Patients With Head and Neck Cancers. Int. J. Radiat. Oncol..

